# Face and content validation of the amyotrophic lateral sclerosis—Bulbar dysfunction index (ALS-BDI)

**DOI:** 10.3389/fneur.2022.1078612

**Published:** 2023-01-05

**Authors:** Yana Yunusova, Ashley Waito, Carolina Barnett Tapia, Anna Huynh, Rosemary Martino, Agessandro Abrahao, Gary L. Pattee, James D. Berry, Lorne Zinman, Jordan R. Green

**Affiliations:** ^1^Department of Speech-Language Pathology, Rehabilitation Sciences Institute, University of Toronto, Toronto, ON, Canada; ^2^Hurvitz Brain Sciences Research Program, Sunnybrook Research Institute, Toronto, ON, Canada; ^3^KITE – University Health Network, Toronto, ON, Canada; ^4^Division of Neurology, Department of Medicine, University of Toronto and University Health Network, Toronto, ON, Canada; ^5^Dalla Lana School of Public Health, Institute of Health Policy, Management, and Evaluation, University of Toronto, Toronto, ON, Canada; ^6^Department of Otolaryngology – Head and Neck Surgery, University of Toronto, Toronto, ON, Canada; ^7^Krembil Research Institute, University Health Network, Toronto, ON, Canada; ^8^Department of Medicine, Division of Neurology, Sunnybrook Health Sciences Centre, Toronto, ON, Canada; ^9^Neurology Associates, P.C., Lincoln, NE, United States; ^10^Sean M. Healey and AMG Center for ALS, Massachusetts General Hospital, Boston, MA, United States; ^11^Department of Communication Sciences and Disorders, MGH Institute of Health Professions, Boston, MA, United States; ^12^Speech and Hearing Bioscience and Technology, Harvard University, Boston, MA, United States

**Keywords:** bulbar ALS, assessment, development, ALS-BDI, COSMIN, rehabilitation

## Abstract

**Purpose:**

Early detection and tracking of bulbar dysfunction in amyotrophic lateral sclerosis (ALS) are critical for directing management of the disease. Existing physiological assessments of bulbar dysfunction are often inaccessible and cost-prohibitive for clinical application. Existing clinical assessments are limited. The overall goal of our research is to develop a brief and reliable, clinician-administered assessment tool, the ALS Bulbar Dysfunction Index (ALS-BDI) to evaluate bulbar dysfunction. The aim of this study was to establish content and face validity of the ALS-BDI through item generation and reduction, including item scoring.

**Methods:**

The design of the ALS-BDI followed guidelines outlined by the COnsensus-based Standards for the selection of health Measurement INstruments (COSMIN). The design stage of the ALS-BDI involved two steps: (Step 1) the generation of candidate items from a literature review of commonly used clinical tools, and selection of items following a review of item reliability and item relevance and expert consensus; (Step 2) the assessment of their content and face validity *via* online survey feedback from experts (*n* = 35). The initial design was followed by a semi-structured cognitive interview with Speech-Language Pathologists (*n* = 5) to finalize a testable draft of the instrument.

**Results:**

Two drafts of the ALS-BDI were developed. The first draft contained 48 items, after a review of existing clinical tools for their relevance to bulbar dysfunction in ALS. Of the 48 items, 35 items were retained after surveying experts and clinician users for their relevance, feasibility, interpretability, and appropriateness. The second draft of the ALS-BDI contained 37 items, due to one item splitting, based on users cognitive interviews.

**Conclusions:**

The ALS-BDI described in this study aims to provide a brief and reliable, clinician-administered assessment tool to evaluate bulbar dysfunction in patients with ALS. Future research will evaluate the psychometric properties of this tool including its reliability, validity, and responsiveness to change over time.

## Introduction

Amyotrophic lateral sclerosis (ALS) is a fast-progressing neurodegenerative disease affecting upper and lower motor neurons, as well as extramotor (e.g., cognitive-linguistic) brain pathways. The degeneration of motor neurons results in progressive muscle weakness, atrophy, and eventual paralysis. More than 80% of individuals diagnosed with ALS will experience bulbar dysfunction, problems with speaking and swallowing, either at the onset of the disease or with its progression (1–3). The presence of bulbar dysfunction is associated with a more rapid disease course, an overall more debilitating disease presentation, and shortened survival (4). From the patients' perspective, speech impairment and eventual loss of speech are among the worst consequences of the disease (5, 6).

Despite the devastating consequences of bulbar dysfunction on survival and quality of life, and the substantial need to track bulbar changes early and continuously during disease progression, there are currently no validated clinical tools designed solely for this purpose. In a recent comprehensive review of the available tools for assessing bulbar dysfunction in ALS, we particularly noted a scarcity of validated clinician-administered tools (7). The primary means of bulbar assessment in the ALS clinic remains a comprehensive symptom checklist - the ALS Functional Rating Scale-Revised (ALSFRS-R) (8, 9). It includes a total of three questions related to bulbar dysfunction, targeting speech, swallowing, and salivation. ALSFRS-R is typically administered by clinicians but can also be self-administered by patients (10). Speech-language pathologists (SLPs) administer several well-established standardized tests of speech and swallowing including the Frenchay Dysarthria Examination [FDA-2 (11)], the Oral Speech Mechanism Screening Examination (12), or the Clinical Examination of Swallowing in Adults (13), but these tests are not typically used in ALS clinics and clinical trials for several reasons: (1) they do not selectively target the unique neuromotor manifestations of ALS, which include upper motor neuron and lower motor neuron signs and symptoms–and (2) they have not been validated or assessed for their psychometric properties in the context of ALS. As such, current practices of bulbar assessment in ALS clinics remain idiosyncratic, piecemeal, and not well-standardized. The development of more efficient and effective outcome measures continues to be a top research priority in ALS (14).

### Conceptualization of bulbar dysfunction and its assessment domains

**Figure 1:**
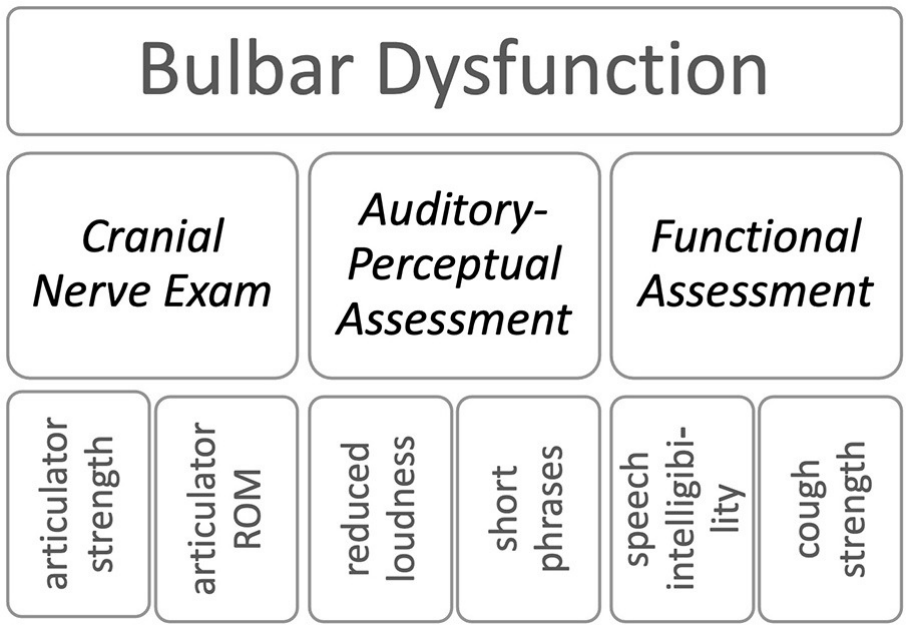
The domains and examples of items of ALS-BDI.

To address this need for an assessment of bulbar dysfunction, thirty experts in neurology, speech-language pathology, and measurement science from the Northeastern ALS Consortium (NEALS) Bulbar Subcommittee met as a group in Boston, USA and engaged in a focus group discussion to identify (1) the limitations of current practices for assessing bulbar function (speech and swallowing) used in clinical and research/ clinical trial settings [see Pattee et al. (15)] and (2) strategies to address these limitations. At the onset, they agreed on the definition of the construct of interest – the bulbar dysfunction in ALS - and the key domains for its assessment. The group conceptualized bulbar dysfunction in ALS *via* the combined status of oromotor structures that (1) underly the phonatory, respiratory, resonatory, and articulatory subsystems of speech as well as prosody, and (2) support the generation of intelligible speech and safe swallowing functions (see example in Figure 1).

The focus groups identified the following three core assessment domains of bulbar dysfunction in ALS: (1) a *Cranial Nerve Exam;* (2) an *Auditory-Perceptual Assessment* [of speech and voice]; and (3) *Functional Assessment* [of overall speech intelligibility, swallowing, chewing, and coughing]. The cranial nerve exam is used in ALS to localize and determine the extent of upper vs. lower motor neuron involvement by testing reflexes, muscle force, range of motion, speed, and symmetry of orofacial musculature (16). Auditory-perceptual assessments rely on clinical expertise to detect and characterize abnormal voice and speech features. The profile of abnormal features is used to document the type and severity of speech impairment and provide supplemental information for confirming disease diagnosis, disease staging, pattern and focality of spread, and underlying neuropathological mechanisms (17). The functional assessment aims at documenting the impact of the neurological damage on functions such as speech (i.e., speech intelligibility) and swallowing (i.e., swallowing safety and efficiency, chewing, and cough) (3, 18–23). We used these consensus findings as the foundation to inform the development of the ALS Bulbar Dysfunction Index (ALS-BDI).

### Conceptual framework for measurement: A formative model

Based on the expert discussion of the conceptual domains for assessment (24), ALS-BDI used a formative (rather than reflective) development model to represent the relationships between potential items and the overall construct (i.e., bulbar dysfunction) to be measured. Within a formative model, all items provide an indication of the construct, but the change in the construct may not necessarily affect all items (25–27). As all items are representative of the overall construct, they are not interchangeable, even if the items are found to be correlated (28). The decision to use a formative model was made because different physiological subsystems can have different patterns of change in ALS (29), but these changes would still be measuring bulbar dysfunction. For example, voice and articulatory disorders may both change substantially over time, but these changes may follow different trajectories during disease progression. Formative measures, as opposed to reflective measures, are not assessed with respect to internal consistency and item-total correlations such as Chronbach's alpha, factor analysis, or the item response theory. The decision to retain or reduce items is first based on their clinical relevance in defining the construct (i.e., content and face validity), then on their reliability, and, finally, on their relationship to other validated measures and responsiveness to change over time (25).

The long-term goal of this research is to develop a reliable and psychometrically validated clinician-administered assessment tool of bulbar dysfunction that would be efficient, standardized, clinically feasible, comprehensive, and responsive to change over time. Our process closely adheres to standards for developing outcome measures described in the COnsensus-based Standards for the selection of health Measurement Instruments [COSMIN, (26)]. The current study addressed the initial stage of the tool development, which aimed to establish the content and face validity of the overall tool and individual test items. To achieve this goal, we (1) systematically identified a large pool of candidate test items based on prior research and (2) empirically determined, through expert opinion, which candidate items to retain. These efforts produced a 37-item draft of ALS-BDI ready for further psychometric testing (i.e., reliability, validity, and responsiveness to change over time).

## Methods

This study was approved by Research Ethics Boards at both data collection sites: Sunnybrook Health Science Centre (Toronto, Canada) and Mass General Brigham (Boston, United States). All study participants provided written informed consent in accordance with the Declaration of Helsinki.

### Study design

COSMIN guidelines were used to inform item generation and item reduction, and then the revision of the ALS-BDI prototype (26). Item generation and item reduction were iterative processes that began with a comprehensive literature review (Step 1). Face and content validity for each item was then assessed by distributing online surveys to experts; following the responses from experts, cognitive interviews were conducted to revise the tool instructions for clarity (Step 2).

#### Step 1: Candidate item generation

The work on this step was undertaken by the core ALS-BDI Development Team, which included authors YY, JG CT, RM, AA, GP, and LZ; all members had substantial experience (>10 years each) working as clinicians (SLPs or neurologists) and as researchers in areas such as ALS, neurodegenerative diseases, motor speech disorders, and swallowing disorders.

First, a literature review of existing tools was conducted to identify candidate items that were representative of the bulbar dysfunction domains. A consideration of the existing tools was essential for ensuring that the tool was consistent with current assessment practices in SLP, resulting in good up-take by clinicians in the future. Relevant tools were identified among the tools used by SLPs to evaluate oromotor deficits, dysphonia, dysarthria, and dysphagia in neurological populations. Candidate items were further selected based on evidence of item reliability and relevance to bulbar ALS established in the research literature (13, 30, 31). Items were removed if they had very low reliability (i.e., kappa <0.45) (32). This lower reliability threshold was considered acceptable for generating a comprehensive initial pool of candidate test items. Items were considered to be (1) highly relevant to bulbar ALS, (2) indicative of UMN or LMN damage, and (3) diagnostic value in ALS based on the literature (16, 24). Items of high relevance to bulbar ALS, even if they were known to have lower reliability (e.g., interpretation of jaw jerk reflex), were retained for further testing in Step 2.

#### Step 2: Face and content validity of initial item set

A test has face validity when its items reflect, according to expert impression, the concept that the test attempts to measure, while content validity is achieved when test items adequately (and thoroughly) capture the construct (domains) of interest, as based on expert opinion (33). We tested the face and content validity of the tool by gathering expert feedback regarding preliminary candidate items collected in Step 1, using (1) an expert survey and (2) conducting cognitive interviews.

### Participants and recruitment

Participants for the expert survey were neurologists and SLPs, who regularly provide services to individuals with the bulbar form of ALS, in a context of multidisciplinary ALS clinics. MND/ALS experts were identified and recruited through the network of contacts at NEALS. These contacts included MND/ALS experts from Canada, USA, and United Kingdom. SLPs were also recruited through a dedicated closed and private group on social media for SLPs working with patients with ALS.

Participants for the cognitive interviews were SLPs who had participated in the survey. We focused solely on SLP's in this sub-step as the primary future user group for ALS-BDI to seek their feedback on the tool as a whole (e.g., order of items relative to the typical flow of assessment, fluency in administration, wording, etc.) (33).

#### Expert survey

To capture expert feedback on the ALS-BDI and scoring scales, we distributed an online survey. An online survey was selected because of its practical capabilities to collect large amounts of data systematically and conveniently. Respondents who agreed to participate in the survey self-identified their clinical expertise (i.e., in neurology or speech-language pathology) when they expressed an interest in the study. Respondents were sent an individualized survey link requesting their demographic information and a subset of ALS-BDI items for feedback.

Neurologists responded exclusively to the items in the *Cranial Nerve Exam* domain, based on their specialization in neurological assessment. SLPs were assigned items across all domains (i.e., *Cranial Nerve Exam, Auditory-Perceptual Assessment, Functional Assessment*), based on their specialization. Items in the Auditory-Perceptual domain were split into two groups in the expert survey to ensure the completeness of responses on all items. As such, we intended to recruit more SLPs than neurologists in total.

We collected quantitative data (ordinal ratings) to gather an impression of the ALS-BDI and qualitative data (free-text responses) to inform the revised draft of this tool. Survey respondents rated each item on a series of 5-point Likert scales in terms of their relevance to the assessment of bulbar dysfunction in ALS, clinical feasibility, interpretability, and clarity of wording, with higher scores indicating higher clarity or relevance. Respondents also indicated whether the ALS-BDI scoring scale was appropriate for each item (i.e., Yes/No). After they had reviewed their assigned subset of items, respondents were given an opportunity to review and provide open-ended feedback on the full list of items, grouped within their subdomains, and propose additional items, if necessary, that would fit within the construct of bulbar dysfunction.

#### Cognitive interviews

Once items were identified through the survey, we compiled the tool in a paper form, including the instructions for administration and scoring and distributed it to SLPs in preparation for cognitive interviews. Cognitive interviewing is a qualitative method that examines how respondents interpret questions and form answers to questions (34). In the context of the assessment item development, cognitive interviewing was primarily used as a pretest method to find and correct any problems with a specific assessment item/scoring criteria (i.e., respondent difficulty when answering a question) (35). When possible, SLPs were also asked to trial the ALS-BDI tool with their patients (with patient consent). During the interviews, we asked SLPs to describe how they would administer each task or item, what each scoring level would mean for them, and how each item corresponded to what they typically do within their clinic routine, using a “think aloud” approach to reveal their thought processes [(34, 35) pp. 42-65]. This process enabled us to identify problematic questions and responses on the ALS-BDI (e.g., multiple or inconsistent interpretations) and revise the tool to be compatible with a clinician's workflow. Respondents were encouraged to be honest in their responses, as their feedback was important to inform the development of a tool that was feasible and practical for clinician's use.

The interviews with SLPs were conducted by a trained researcher based on a detailed interview guide. The interviewer [AW] completed her PhD in speech-language pathology with approximately seven years of experience as an SLP. As an SLP, she was considered an expert in this topic because she shared similar clinical experiences with the SLP respondents and could ask relevant follow-up questions about clinical workflow (36). The interviews were to take place either in-person, over the phone, or through an internet-connected voice over IP (VoIP) service such as Skype. The interviewer took detailed pen and paper notes during each interview. These notes were then expanded immediately after each interview with additional details.

#### Data analysis

For the expert survey, we analyzed the distributions of ordinal ratings using the median rating and interquartile range. Items with a median relevance of less than three were flagged for reduction. Feasibility and interpretability ratings were used to inform edits and improvements, but they were not used to inform reduction if relevance was rated highly.

The method used to analyze the interviews was informed by the qualitative approach described by Willis (35). This approach involved three steps, which were conducted by one team member (AW): (1) review and document individual interviews; (2) compile results across interviews; and (3) write an organized testing report. The qualitative data were entered into a Microsoft Excel spreadsheet and grouped by items. An inductive coding process was performed by identifying content words from comments and extracting them as keywords. The key words were then sorted manually by dragging and dropping them into different categories. Key words that could not be semantically linked to at least one item were moved to a “general category” and subsequently given a more specific label based on the semantic relevance of the key words (e.g., time, tolerance, patient experience). Feedback related to the removal or retention of items, scaling, and wording changes were then summarized by a team member (AW) into a slide deck and organized into different themes to inform the assessment revision process (e.g., item definitions, item scoring, and structure of tool). This slide deck was then presented to the ALS-BDI Development Team for discussion.

#### Revision process

The entire development team participated in a series of meetings focused on reviewing the expert feedback and discussing suggestions for revisions at each stage. All decisions were made based on the group consensus to generate unanimous agreement. These meetings were organized into two parts: reviewing specific items for inclusion/exclusion and then discussing suggestions for improvement based on the feedback themes identified from the expert survey and cognitive interviews. Members voted to accept or reject each suggestion and provided a rationale for their opinions or alternative suggestions based on current literature or their clinical experience. When disagreements occurred, they were resolved by discussions focuses on the item in question's theoretical relevance to bulbar function assessments, its theoretical responsiveness to bulbar ALS, and its potential for being addressed in future studies.

## Results

### Step 1—Candidate item generation

**Figure 2:**
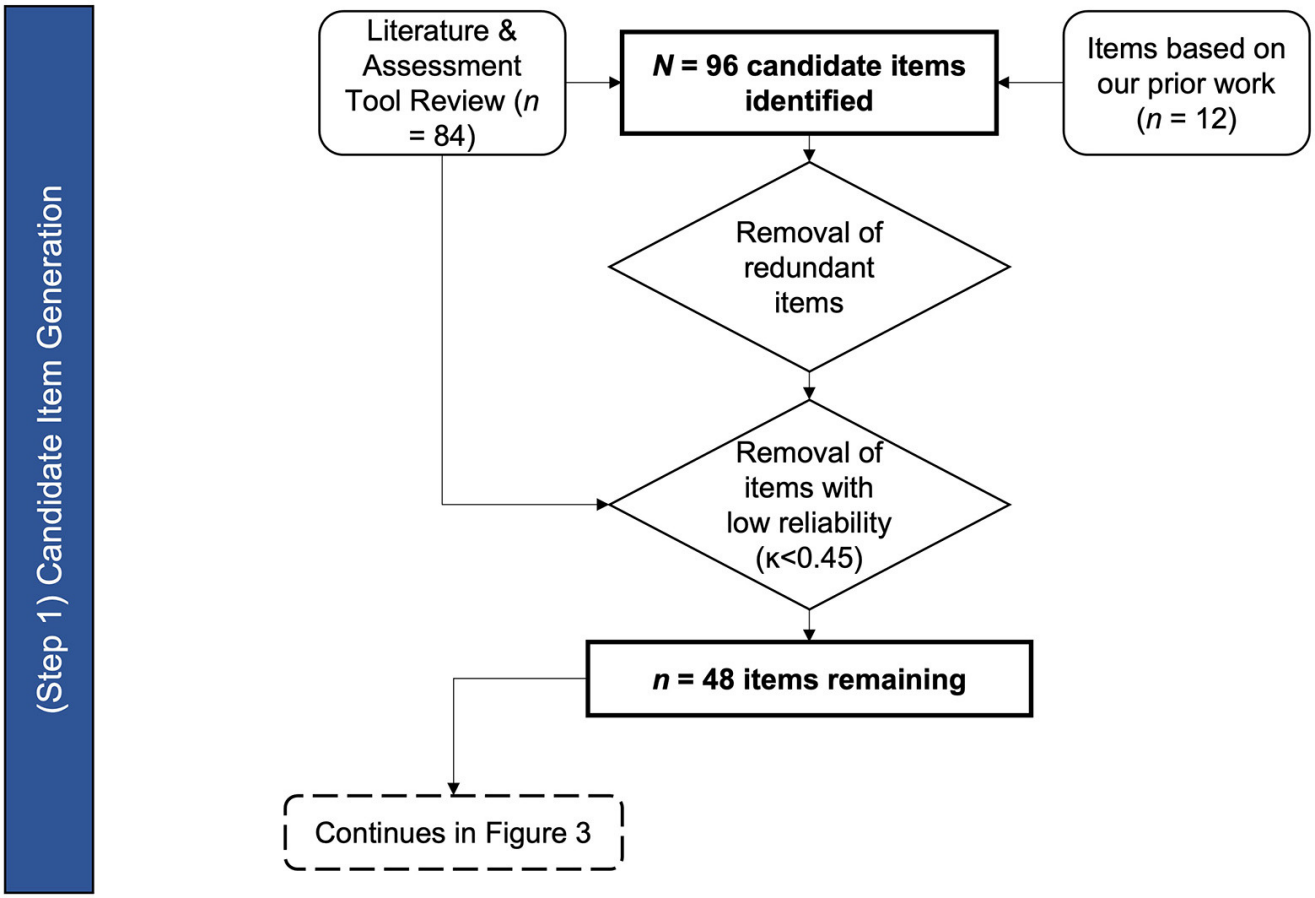
Flow chart of ALS-BDI design stage 1—Candidate item generation.

The result of the existing tools and literature review generated the initial list of candidate items of the ALS-BDI (see Figure 2). The candidate items were selected based on clinical indicators of bulbar dysfunction as assessed during the oral motor exam (OME), auditory-perceptual assessment - organized by physiological subsystems (20), and functional level measures.

Cranial Nerve Exam/OME items were identified based on the review of the Frenchay Dysarthria Assessment (11) (number of OME items = 13); the Oral Speech Mechanism Screening Examination (OSMSE; St. 12) (number of OME items = 15) and the Clinical Examination of Swallowing in Adults (13) (number of OME items = 8). Of these items, six items were deemed reliable in previous psychometric evaluations of these assessments (13, 31). Additional OME items were added based on our previous findings of abnormal articulator range of motions and speeds in bulbar disease (37–41). A “jaw jerk reflex” item was also added based on high relevance to ALS, despite previous concerns about the reliability of this item (42, 43). A total of 16 OME items were included in the first draft of ALS-BDI: 14 of those items assessed the tongue, lips/face (combined) and jaw for weakness, range of motion, speed, atrophy, and fasciculation (excluding jaw fasciculations), and the remaining two items assessed facial and jaw jerk reflexes.

The Auditory-Perceptual Assessment domain of ALS-BDI includes items that capture dysphonia and dysarthria. Dysphonia items were adapted from a well-designed and validated clinical dysphonia assessment instrument, the CAPE-V (44, 45) (number of dysphonia items = 6). Additional dysphonia items – the ability to elevate pitch and pitch breaks – were added based on previous research on ALS (37, 46). A total of eight items reflecting the integrity of the phonatory subsystem were included in the ALS-BDI. The remaining Auditory Perceptual items were taken from the Mayo Clinic Dysarthria Study Dimensions (17). Of the 48 total Dysarthria Dimensions items (excluding overall rate and intelligibility), 15 were identified as most reliable (30) and relevant to ALS. These items were arranged to cover respiratory, resonatory, articulatory, and prosodic controls, commonly affected by bulbar disease across the disease progression (37). One additional item to represent an overall dysprosody was added. Like the original clinical exams that we used for developing our item set, the evaluations in the ALS-BDI were to be based on standard tasks including vowel phonation, DDKs, as well as sentence and passage readings.

The functional exam domain items focused on the assessment of the core functions affected by ALS, namely, speaking, swallowing, chewing, and coughing This enabled evaluation of bulbar deficit severity. The following items captured these functions: auditory-perceptual ratings of dysarthria severity, dysfluency, speaking rate, speech intelligibility, groping, and voluntary cough strength; 3 oz of water swallow test; and chewing duration test. These items have yielded important diagnostic information in bulbar ALS based on literature (18, 22, 23, 29, 47). Although the perceptual evaluation of cough strength and reliability raised concerns (48), all four items were retained based on their high relevance to ALS.

In summary, a comprehensive search of relevant literature yielded 84 candidate items. Twelve additional items came from reviewing our prior findings on bulbar ALS, bringing the total to 96 candidate items. Forty eight items were removed due to lack of relevance to construct, poor reliability, or redundancy, and 48 items (16 in Cranial Nerve Exam, 24 in Auditory-Perceptual Assessment, and 8 in the Functional Assessment domains) were retained for the next stage of the ALS-BDI's development (see Appendix SA1 for the list of items).

### Step 2—Face and content validity of the item set

**Figure 3:**
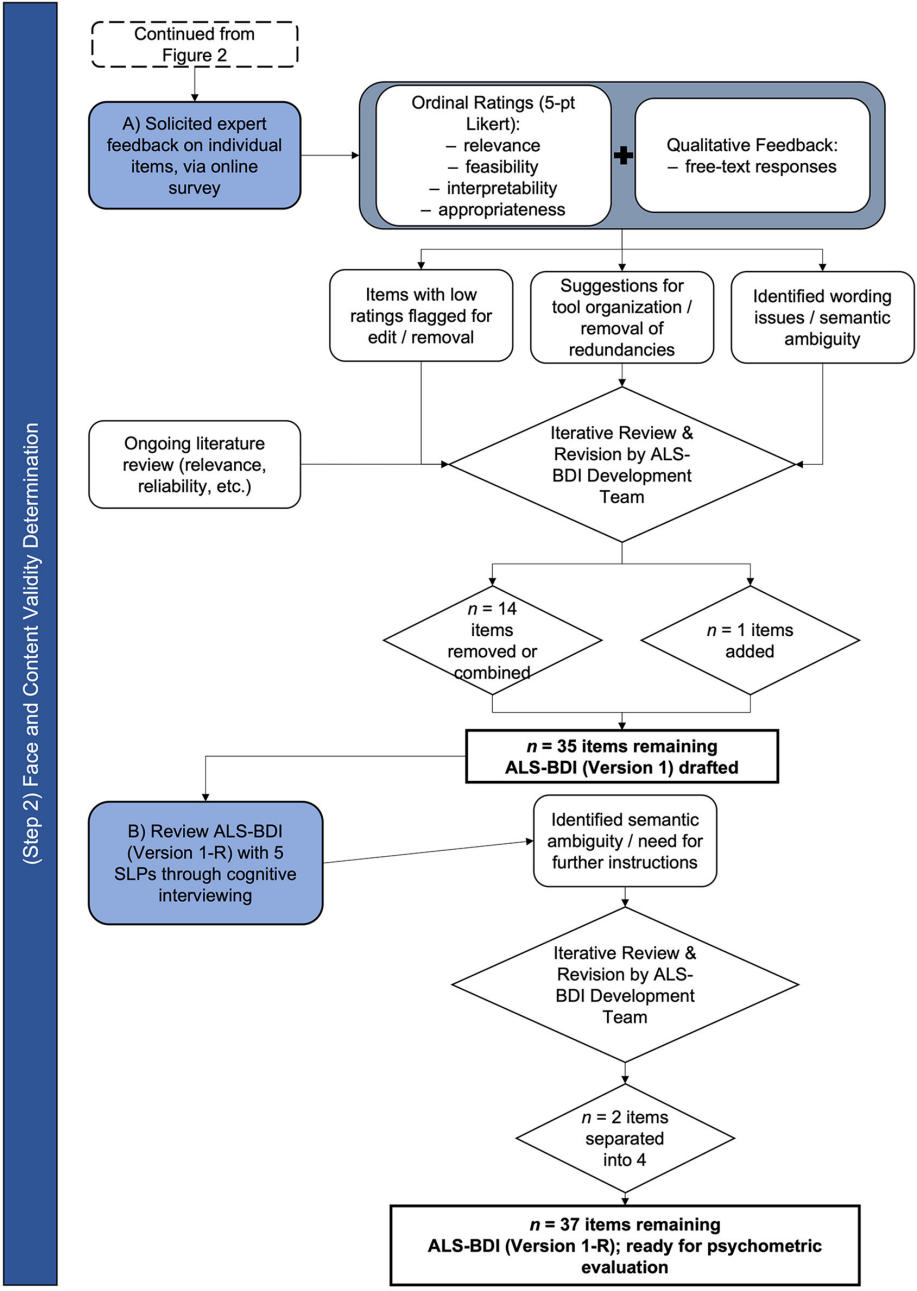
Flow chart of ALS-BDI design stage 2—Face and content validity determination.

The process of establishing the face and content validity of ALS-BDI is schematically represented in Figure 3.

#### Expert survey

Survey recruitment and response rate to the survey are presented in Table 1. Overall, we achieved a 71% response rate for survey completion. Demographic details for 35 survey respondents (28 SLPs and 7 neurologists) are summarized in Table 2. All respondents had prior clinical experience working with individuals with ALS.

**Table 1:** Survey response rate by subtest.

**Domain**	**Total number of links provided**	**Surveys completed**	**Incomplete/ No response**	**Response rate**
Cranial nerve exam (16 items)	17	10	7	59%
Auditory-perceptual assessment (phonatory, respiratory; 12 items)	10	7	3	70%
Auditory-perceptual assessment (resonatory, articulatory, prosody; 12 items)	11	9	2	82%
Functional assessment (eight items)	11	9	2	82%
Total	49	35	14	71%

**Table 2:** Demographic details of survey respondents.

		**SLPs (%) *n* = 28**	**Neurologists (%) *n* = 7**
Sex	Female	27 (96)	1 (14)
	Male	1 (4)	6 (86)
Age	25–44 years	18 (64)	2 (29)
	45+ years	10 (36)	5 (71)
Experience working with individuals with ALS	≤ 10 years	20 (71)	2 (29)
	5–10 years	8 (29)	5 (71)

SLP, speech language pathologist; ALS, amyotrophic lateral sclerosis.

Most items (77%) included on the ALS-BDI were rated from “somewhat” to “highly relevant” to the evaluation of bulbar dysfunction in ALS. No items were rated as “highly irrelevant.” In terms of feasibility and interpretability, all items that were rated as “highly relevant” were also rated to be “somewhat” or “extremely easy to test” in a clinical setting, and 91% of items were rated as “clear, easy to understand”; respondents indicated that the remaining (i.e., unclear) items would require “minor changes to improve wording.” A table of ratings from the expert panel survey is provided in Appendix SA1.

Respondents provided an abundance of constructive feedback to improve the clarity and structure of the ALS-BDI and optimize its practical utility in a clinical setting. Specifically, they indicated, which instructions and scoring required clarification, and provided suggestions to improve the overall workflow of the tool.

Based on the feedback of survey respondents, we removed eleven items, combined three items with other items, and added one item (i.e., DDK rhythm). Following these revisions, 35 items were included on the ALS-BDI for further testing. To improve instructions for scoring, we clarified definitions for each item on the ALS-BDI at each level of severity.

#### Cognitive interviews

Five SLPs (four females) agreed to participate in cognitive interviews to assist with clarifying instructions for the ALS-BDI. All SLPs were from North America (fourCanada, one USA) and Caucasian. Two of the SLPs trialed the ALS-BDI with a patient at their site prior to their interview. Four SLP interviews were conducted over the telephone (*n* = 1) or *via* Skype (*n* = 3); one interview was conducted in person. On average, interviews took between 1 to 1.5 h to complete. The interviews illuminated ambiguity within several instructions and/or definitions, including two “double-barrelled” questions (i.e., combined two responses in one) (49). Respondents also identified where the order of items misaligned with the logical flow of a clinical appointment (e.g., overall speech intelligibility estimate to be moved forward in the test flow).

Based on these results and additional feedback from the SLPs, we reordered several tasks and grouped related items into practical categories (e.g., all “swallowing items” were grouped); we separated DDKs into alternating (i.e., repeat same syllable, each for “puh,” “tuh,” and “kuh”) and sequential (i.e., repeat syllable sequence “puh-tuh-kuh”) motion rates; and we made minor changes to wording, as needed, to eliminate ambiguity. The result was a second version of the tool with 37 items in total. The two first items represented Functional Speech Severity group, judged initially and globally based on an intake conversation with a clinician; they were followed by 10 items representing Cranial Nerve Examination; 22 items were included in the Auditory-Perceptual Assessment representing each physiological speech subsystem affected by ALS, including six phonatory, four respiratory, four prosodic, one resonatory, and seven articulatory items. “Dysphagia screen” at the end of the tool included ratings of three items - voluntary cough strength, the 3 oz water test, and chewing duration.

When asked about the tool's relevance to their clinical practice in ALS, the interviewed SLPs reiterated that the assessment domains and individual items of ALS-BDI were consistent with current practice aims in ALS care. The standardized instructions and item-specific scoring guide, implemented according to feedback from the survey, were found to be helpful in the administration. Those SLPs who trialed the ALS-BDI in their clinical practice noted that the patients to whom ALS-BDI was administered did not experience or express any concerns with the tool's administration.

## Discussion

Current approaches to clinical practice and service delivery are idiosyncratic to individual clinics, poorly validated, lengthy, subjective, rely heavily on symptom checklists, and do not perform well either for detecting bulbar impairment or for monitoring disease progression (7). A consensus group of clinicians and scientists, members of NEALS Bulbar Subcommittee, established the need for a standardized and validated clinician-administered bulbar motor assessment tool (15). In response to this need, we have been developing the ALS-BDI, a clinician-administered assessment of bulbar function in ALS that spans the three core assessment domains that capture the concept of bulbar dysfunction (i.e., Cranial Nerve Exam, Auditory-Perceptual Assessment, and Functional Assessment). The aim of the tool was to focus solely on motor dysfunction, as there have been recent excellent developments in instruments that assess the cognitive-linguistic deficits associated with ALS (50). The current report described the two-stage process used (1) to generate and screen test items and (2) to establish their content and face validity. The outcome of this effort was the version of the ALS-BDI (Version 1, Revised)—a 37 item assessment that has been vetted for its relevance, feasibility, administrative clarity, interpretability, appropriateness, and reported reliability.

The initial item pool based on the review of the existing SLP tools and research literature contained 96 candidate items. Generation of the initial item set was significantly facilitated by the availability of existing tools for evaluating oromotor function, dysphagia, voice, and speech (11, 13, 17, 44), with most of these tools having multiple test items that were relevant, based on the definition of bulbar dysfunction domain as defined by NEALS bulbar experts. This approach to generating the pool of candidate test items was efficient in that most of the items were rated at least “somewhat relevant” (77%) and “easy to understand” (91%). However, over half (i.e., 48 of the 96) of the items were eliminated after being labeled unreliable (based on extant literature), redundant, and/or irrelevant. The cognitive interviews that followed were critical for further refining the item pool (i.e., eliminating, adding, and combining items), and improving the wording of the instructions. The entire process was overseen by the ALS-BDI Development Team, who, at each step, responded to expert feedback and revised the tool iteratively to produce its final testable version.

Although we succeeded in developing a testable version of the tool for the assessment of bulbar dysfunction, there were some potential limitations in our approach. Specifically, at this stage, the number of neurologists who participated in the development was relatively small. We realize that there is a need to develop a tool that can be administered by various ALS specialists, not only SLPs. However, currently, the tool contains a substantial number of items that require specialized training in the auditory-perceptual rating of subtle speech features such as nasality or prosody. Few professions other than SLP provide the level of training needed to discern these features. However, once the number of items gets reduced in item reliability/ validation testing, we intend to examine the feasibility of developing a simple training module that would prepare assessors without an SLP background to evaluate these items accurately. Until this happens, the feedback from other disciplines (i.e., neurology) is limited to the items of the cranial nerve exam.

The long-term goal of this research is to develop a psychometrically validated clinician-administered bulbar assessment tool based on the COSMIN guidelines. The work described in this report defined Cycle 1 of the assessment tool development and focused on generating a first draft of a tool with established item and overall tool's content and face validity. Although content and face validity are important, they are insufficient to develop an outcome measure. During the next development cycle, Cycle 2, we will examine the test-retest and interrater reliability of individual items and of the overall tool, and additional items will be removed from the pool if unreliable. During the last development cycle, Cycle 3, we will establish construct validity with respect to clinical gold standards (i.e., ALFRS-R total and bulbar subscore) and determine the tool's responsiveness to change with disease progression, alongside the minimally important difference and minimal detectable change [see protocol description in Yunusova et al. (51)].

At the end of this process and to the best of our knowledge to date, the ALS-BDI will be the first standardized and psychometrically validated clinician-administered bulbar assessment tool. The ALS-BDI aims to meet the key requirements for developing new assessment tools, namely, (1) supported by the multidisciplinary ALS expert consensus; (2) efficient, ALS-specific, and standardized administration protocol; and (3) reliable, valid, responsive, and clinically meaningful. The broad long-term impact of the ALS-BDI will include improving detection of bulbar ALS, expediting diagnosis, improving clinical decision-making, and accelerating ALS clinical trials and drug discovery. Because the instrument is consistent with current assessment practices in the field of speech-language pathology, which are based on clinician ratings of speech, swallowing, and oral structure and function, it is likely to have up-take within clinical practice settings focused on ALS.

## Data availability statement

The raw data supporting the conclusions of this article will be made available by the authors, without undue reservation.

## Ethics statement

The studies involving human participants were reviewed and approved by Research Ethics Boards at both data collection sites: Sunnybrook Health Science Centre, primary (Toronto, Canada; ID3080) and Mass General Brigham (Boston, United States; #2013P001746). The patients/participants provided their written informed consent to participate in this study.

## Author contributions

YY, CB, RM, AA, LZ, JB, GP, and JG conceived the study and prepared the funding application. AW performed data collection and analyses. YY and AW wrote the manuscript. All listed authors meet authorship criteria and that no others meeting the criteria have been omitted, contributed to the design of the study protocol, reviewed feedback and came to consensus on tool design, read, edited, and approved the final manuscript.
