# Supplementary material for: Face and content validation of the amyotrophic lateral sclerosis—Bulbar dysfunction index (ALS-BDI)
Source: Front Neurol. 2023 Jan 5;13:1078612. doi: 10.3389/fneur.2022.1078612 (PMC9849694; doi:10.3389/fneur.2022.1078612)
Supplement: Supplementary file 1 [file Table_1.docx]

*APPENDIX SA1. Quantitative results from expert survey. Items with median relevance < 3 were flagged for removal. Items with wide range of ratings between respondents (i.e., IQR ≥ 1.5) were flagged for priority review and potential removal.*

|  |  | **Relevance** | | **Feasibility** | | **Interpretability** | | **Responses Appropriate for Item**  **(% “Yes”)** |
| --- | --- | --- | --- | --- | --- | --- | --- | --- |
| **Item** | **Name** | **Median** | **IQR** | **Median** | **IQR** | **Median** | **IQR** |  |
| 1 | Tongue strength | 5 | 0.75 | 4 | 2.25 | 4 | 1 | 90 |
| 2 | Tongue atrophy | 5 | 0 | 4.5 | 1 | 5 | 0 | 80 |
| 3 | Tongue symmetry | 3 | 2 | 4 | 1.5 | 4 | 1.75 | 60 |
| 4 | Tongue fasciculations | 5 | 0.75 | 4 | 1.75 | 5 | 0.75 | 80 |
| 5 | Tongue ROM | 4 | 1.75 | 3.5 | 2 | 5 | 0.75 | 90 |
| 6 | Tongue speed of motion | 5 | 0.75 | 5 | 2.5 | 5 | 2.5 | 90 |
| 7 | Lip/face strength | 5 | 1.75 | 4 | 0.75 | 4 | 1 | 90 |
| 8 | Lip/face symmetry | 3 | 2.25 | 4 | 1.5 | 4 | 1.5 | 90 |
| 9 | Lip/face ROM | 2.5 | 2 | 3 | 2 | 4 | 2.25 | 80 |
| 10 | Lips speed of motion | 3.5 | 2.25 | 3.5 | 2 | 4 | 2.25 | 70 |
| 11 | Facial reflexes | 4.5 | 2 | 4.5 | 2 | 5 | 0 | 50 |
| 12 | Jaw strength | 4 | 1.5 | 3.5 | 3 | 5 | 1 | 70 |
| 13 | Jaw symmetry | 3.5 | 2 | 4 | 2.25 | 4 | 2.5 | 80 |
| 14 | Jaw ROM | 3.5 | 2.25 | 4 | 1.5 | 5 | 1 | 80 |
| 15 | Jaw speed of motion | 3 | 2 | 3.5 | 2.25 | 4 | 1.5 | 90 |
| 16 | Jaw jerk reflex | 5 | 0.25 | 5 | 1 | 5 | 0 | 70 |
| 17 | Strained voice | 5 | 0 | 4 | 1 | 5 | 1 | 86 |
| 18 | Breathiness | 4 | 1 | 4 | 1 | 5 | 0 | 86 |
| 19 | Roughness | 4 | 2 | 4 | 1 | 5 | 1 | 100 |
| 20 | Reduced loudness | 5 | 1 | 5 | 1 | 5 | 0 | 86 |
| 21 | Unusually high/low pitch | 4 | 1 | 4 | 0 | 5 | 1 | 86 |
| 22 | Pitch breaks | 4 | 1 | 4 | 1 | 5 | 1 | 86 |
| 23 | Inability to elevate pitch | 3 | 1 | 4 | 1 | 5 | 0 | 100 |
| 24 | Overall dysphonia | 5 | 0.5 | 4 | 0.5 | 5 | 0.5 | 100 |
| 25 | Short phrases | 5 | 1 | 5 | 1 | 5 | 1 | 86 |
| 26 | Inappropriate pauses | 3 | 1 | 4 | 0.5 | 5 | 1 | 86 |
| 27 | Forced inspiration/ expiration | 4 | 1 | 4 | 2 | 4 | 1 | 86 |
| 28 | Audible inspirations | 4 | 0 | 4 | 1 | 5 | 0 | 86 |
| 29 | Hypernasality | 5 | 0 | 4 | 1 | 5 | 0.5 | 100 |
| 30 | Nasal emissions | 5 | 1 | 4 | 2 | 5 | 0.5 | 89 |
| 31 | Slow rate DDK | 5 | 0.5 | 5 | 1 | 5 | 1.5 | 89 |
| 32 | Imprecise consonants | 5 | 0 | 5 | 1 | 5 | 0 | 89 |
| 33 | Distorted vowels | 4 | 1 | 4 | 1.5 | 5 | 0.5 | 78 |
| 34 | Prolonged phonemes | 4 | 1 | 4 | 0.5 | 5 | 0.5 | 78 |
| 35 | Monopitch | 4 | 1 | 4 | 1.5 | 5 | 0.5 | 89 |
| 36 | Monoloudness | 4 | 2 | 4 | 2 | 5 | 0.5 | 89 |
| 37 | Loudness decay | 5 | 2 | 4 | 1 | 5 | 0 | 100 |
| 38 | Excess and equal stress | 4 | 1.5 | 4 | 2 | 5 | 0.5 | 78 |
| 39 | Reduced stress | 4 | 1.5 | 4 | 2.5 | 5 | 1 | 78 |
| 40 | Overall dysprosody | 4 | 1 | 4 | 1 | 5 | 1 | 89 |
| 41 | Dysarthria severity | 5 | 0 | 5 | 0 | 5 | 0 | 89 |
| 42 | Dysfluency | 2 | 2 | 5 | 1 | 5 | 0 | 78 |
| 43 | Speaking rate | 5 | 0 | 5 | 0 | 5 | 0 | 67 |
| 44 | Groping & Speech Errors | 2 | 2 | 4 | 3 | 5 | 1.5 | 78 |
| 45 | Speech intelligibility | 5 | 0 | 5 | 1 | 5 | 1 | 78 |
| 46 | Chewing time | 5 | 1 | 4 | 2 | 5 | 1 | 89 |
| 47 | 3oz water swallow | 5 | 0.5 | 5 | 0 | 5 | 0 | 78 |
| 48 | Voluntary cough | 5 | 1 | 5 | 0.5 | 5 | 0.5 | 78 |
